# “Sometimes it can be like an icebreaker”: A mixed method evaluation of the implementation of the Refugee Health Screener-13 (RHS-13)

**DOI:** 10.1016/j.jmh.2024.100243

**Published:** 2024-07-15

**Authors:** Ana Hagström, Henna Hasson, Anna-Clara Hollander, Carl Vahtra, Sara Delilovic, Hanna Augustsson

**Affiliations:** a Region Stockholm, Centre for Epidemiology and Community Medicine (CES, with Swedish acronym), Sweden; b Department of Learning, Informatics, Management and Ethics, Karolinska Institutet, Sweden; c Department of Global Public Health, Karolinska Institutet, Sweden

**Keywords:** Forced migrants, Mental health screening, RHS-13, Health assessment, CFIR

## Abstract

**Background:**

Forced migrants are at risk of developing mental illness, yet challenges remain with underutilization of mental healthcare among this population. This study examined the implementation of the Refugee Health Screener-13 (RHS-13) in the health assessment for forced migrants in eight primary health care centres in Stockholm Region, Sweden.

**Methods:**

A mixed-methods convergent parallel design was used, combining nurses self-reported quantitative data on the levels and reasons for RHS-13 use in the health assessment with qualitative interview data on the barriers and facilitators for RHS-13 use. The Consolidated Framework for Implementation Research (CFIR) was used as a coding framework for the qualitative analysis.

**Results:**

Levels of RHS-13 use varied between primary health care centres, resulting in two groups: three centres with high-level (65–92%) and five centres with low-level (0–36%) implementation. Factors related to the tool itself, as well as the inner and outer context, influenced the use of RHS-13. Language barriers, insufficient time, and lack of trust in the validity and utility of RHS-13 were the main barriers, while its availability in many languages and that it was perceived as an important complement to the health assessment were the main facilitators.

**Conclusion:**

RHS-13 contributes to the standardization of assessing mental health in the health assessment. Identifying context-based implementation strategies and addressing language and time issues as well as nurses trust in the tool's utility are recommended to enhance the use of RHS-13.

## Introduction

Forced migrants – asylum seekers, refugees, and individuals fleeing from war or persecution – have a high risk of developing mental illness such as depression, anxiety, or post-traumatic stress disorder (PTSD) (Blackmore et al., 2020; Carroll et al., 2023; Li et al., 2016; Magwood et al., 2022). Exposure to potentially traumatic events before and during their migration, the asylum process itself, and acculturation upon resettlement, are all risk factors for mental illness and psychosocial distress; thus, mental health assessments are recommended upon resettlement (CDC 2015; National Board of Health and Welfare Socialstyrelsen, 2011). Despite being at higher risk, challenges remain in accessing mental healthcare and in assessing mental illness among forced migrants (Hollander et al., 2020).

### Mental health assessment

The use of standardized questionnaires when screening for mental illness can facilitate mental-health assessments among forced migrants and contribute to a systematic, coherent, and quantifiable assessment as an alternative to the clinical interview (Magwood et al., 2022; Bagby et al., 2006). For example, studies show that screening instruments can be effective in detecting PTSD in forced migrants (Berthold et al., 2019; Magwood et al., 2023; Hollifield et al., 2013). A tool specifically developed for refugees commonly used in recent years is the Refugee Health Screener-13 (RHS-13) (Hollifield et al., 2016). It has been shown to be user-friendly and time-efficient when assessing symptoms of depression, anxiety, and PTSD in this population (Hollifield et al., 2016; Bjärtå et al., 2018; Delilovic et al., 2023).

Despite this, mental-health screening can be challenging, especially among forced migrants. Challenges include language and cultural barriers, mental-health stigma, determining an acceptable and appropriate screening environment, the need for longer appointment times, and obstacles to referring individuals to secondary care (Sheth et al., 2023; Nickerson et al., 2017; Willey et al., 2020). Moreover, the diversity within the refugee population suggests that universal screening may not be the most efficient strategy (Sheth et al., 2023). Thus, knowledge is still lacking on how to incorporate mental-health screening procedures among forced migrants into routine care, and how contextual and implementation requirements influence the use of such procedures (Magwood et al., 2023).

The aim of this study was to examine the implementation of RHS-13 in health assessments (HAs) for forced migrants carried out by nurses in primary health care centres (PHCCs) in Sweden. The following research questions were investigated: 1) To what level is RHS-13 used in the HA?, 2) What barriers and facilitators influence the use of RHS-13 in the HA?

## Methods

### Study design

This study had a mixed-methods convergent parallel design (Demir and Pismek, 2018) that combined nurses self-reported quantitative data for RHS-13 use with qualitative interview data, deductively analysed using the Consolidated Framework for Implementation Research (CFIR) 2.0 (Damschroder et al., 2022). The study is reported according to the Consolidated Criteria for Reporting Qualitative Research (COREQ) (Tong et al., 2007).

### Setting

In the past decade, Sweden has experienced significant shifts in migration trends. From being a major recipient of non-European asylum-seekers and family reunification migrants, peaking in 2015–2016, policy is shifting towards the European union minimum level (SCB), Statistikdatabasen 2024). Forced migrants include asylum seekers, UNHCR resettled refugees, family reunification migrants, and undocumented migrants, who all have the right to an HA in Sweden (National Board of Health and Welfare Socialstyrelsen, 2011). The Swedish migration Agency (SMA) is responsible for accommodation throughout the asylum process and provides reception centres, although it permits asylum-seekers to stay in private accommodation under certain circumstances (Swedish Association of Local Authorities and Regions, 2022; 994). Consequently, essential services, including HAs, are dispersed among various actors. Resettled refugees are directly granted residency and the municipalities take responsibility for the first period after arrival in Sweden, including booking the HA (Swedish Association of Local Authorities and Regions, 2023).

This study was conducted in the capital region, Stockholm, at PHCCs performing free-of-charge HAs for forced migrants, which are recommended within three months after arrival in Sweden, although in practice many migrants receive the HA after a longer stay. All PHCCs in the region (*n* = 8) performing HAs were invited to participate. These centres are essentially primary PHCCs, but they have the additional responsibility of conducting HAs and offer some care and counselling to asylum seekers. Guidelines stipulate questions on medical history, current physical and mental health status, migration-related concerns, and an introduction to the Swedish healthcare system. HAs structure, implementation, and uptake can differ across regions (National Board of Health and Welfare Socialstyrelsen, 2011). The professionals involved typically include nurses, psychotherapists, psychologists, physicians, and – at some centres – administrators responsible for booking the HAs. The PHCCs organize the work depending on the expected arrival of migrants in the region. Nurses carry out the HAs, and at times this is the nurses’ only work task; during other periods, they combine it with other patient work depending on the number of migrants arriving in the region. In order to provide psychosocial support, some PHCCs dedicate a psychotherapist to these patients, while others refer them to other health services.

In Stockholm, the health authorities allocate migrants to PHCCs providing HAs based on where they are accommodated within the region. Forced migrants, a diverse group from various low- and middle-income countries, have educational backgrounds ranging from illiteracy to university-level degrees. Consequently, some PHCCs more commonly serve specific language groups and literacy levels. Professional interpreters are frequently used. During Covid-19, telephone interpretation became the norm, but prior to that an in-person interpreter during the HA was more common. Following an evaluation of mental health assessments in the HAs (Delilovic et al., 2018) and a pilot in two PHCCs in October 2018–March 2019 (Delilovic et al., 2020), regional health authorities assigned the integration of RHS-13 into the mental health assessment in all PHCC conducting HAs in December 2020. The pilot showed inconsistent RHS-13 implementation, as well as varying levels of acceptance and feasibility among healthcare professionals, which necessitated evaluating the implementation of RHS-13 when it was incorporated in all centres. During the Covid-19 pandemic, when many non-emergency and routine examinations in healthcare were put on pause or digitalized, these HA practices continued as usual.

An educational meeting was held in January 2021 and the implementation period started in February 2021. The centres started implementing RHS-13 at different times due to local needs; however, the implementation was evaluated for a period of six months from their individual start date.

### Implementation strategy

Based on the Expert recommendation for Implementation Change (ERIC) (Powell et al., 2015), the first and last authors identified three types of strategy to facilitate the implementation of RHS-13 in the HAs: an educational meeting, educational materials, and a learning collaborative. These strategies were deemed suitable for the purpose of informing about the innovation and supporting the implementation, and adjusted to the PHCC needs and resources. The first author, with past work experience in HAs, organized the educational meeting with the aim to familiarize nurses conducting HAs with RHS-13, teaching them its usage, interpretation of cut-off scores, and referral processes. The educational meeting also provided information on the prevalence of mental illness among forced migrants and detailed documentation practices for RHS-13 during the implementation period. A few administrators in charge of booking HA appointments also participated. Educational materials were distributed to all nurses to reinforce the training and support effective implementation. One nurse that was on leave of absence in January 2021 and one new nurse that started during the implementation period got an individual educational meeting with same information. During the implementation period, the material was revised according to feedback from the participants. A learning collaborative was formed within an existing local network, where staff from the PHCCs met twice each year to discuss various queries, including RHS-13 issues. The centres that had previously tested RHS-13 in the pilot shared their experiences during the educational meeting and within the learning collaborative, they were also among the eight implementing centres in this study.

### Refugee health screener (RHS-13)

RHS-13 (Bjärtå et al., 2018) is a self-administered questionnaire developed for a refugee population and for individuals over 14 years old, to screen for mental illness. RHS-13 is validated with positivity cut-offs for PTSD, depression, and anxiety in different forced migrant populations (Hollifield et al., 2016; Bjärtå et al., 2018), it is available in 20 languages and commonly used in an international context (Institute, 2024). The questionnaire includes 13 symptom items with instructions to indicate the degree to which these symptoms have been bothersome over the past month, ranging from 0 “not at all” to 4 “extremely”. To account for diverse cultural norms and literacy issues, the developers included symbols of jars containing varying amounts of beans in relation to the response scale of 0–4.

### Participants

Of the 16 nurses who conducted HAs for migrants in Stockholm, 10 (all female) from eight PHCCs agreed to join the study. Four were new to HAs, one was very experienced and knowledgeable, and five had moderate experience.

## Data collection

### Level of RHS-13 use

Throughout the six-month implementation period, nurses documented whether they had used RHS-13 or not after each HA in a form constructed for the purpose. The form had one column for date for HA, a column with screened with RHS-13 yes/no, sex of the RHS-13 user and columns exclusion-criteria (supplementary material 5). If they had not used it, they gave reasons for exclusion. The exclusion criteria were set alternatives based on reasons for exclusion that had transpired when RHS-13 was pilot tested (supplementary material 5).

### Barriers and facilitators

The CFIR 2.0 was used to study barriers and facilitators for the use of RHS-13, and it guided the development of the interview guide. CFIR is a comprehensive framework combining implementation theories focusing on factors that might influence implementation within organizations and workplaces. Five domains capture the context and characteristics of innovation and innovation-users: innovation, outer setting, inner setting, individual characteristics, and implementation process. Semi-structured interviews were conducted with all the nurses (10) after the implementation period, focusing on the barriers and facilitators of the process, and the perceived utility of RHS-13 in the HA. The RHS-13 levels from their PHCC were presented, offering an opportunity for commenting on the level of use.

The interviews were conducted by authors AH (female PhD candidate, RN, MSc) and CV (male, PhD), and most of them were conducted at the informant's workplace by CV. Due to Covid-19 restrictions, two interviews took place via videocall. The interviews lasted 45–70 min and all were audio-recorded and transcribed verbatim.

## Data analysis

### Level of RHS-13 use

The nurses’ reported use of RHS-13 in the HA was compared with the total number of completed HAs per month, according to data obtained from the regional health authorities. We then calculated the proportion of HAs that included RHS-13 for each PHCC. The level of implementation of RHS-13 at each center was evaluated by comparing the frequency of its use against the number HAs conducted monthly. The centers were then categorized as having high or low implementation levels of RHS-13 by discussion among four of the authors (AH, ACH, HA, and HH). No predetermined cut-off levels were used in the assessment. The number of exclusions was summarized.

### Barriers and facilitators

The interviews were analysed using content analysis with a deductive approach (Graneheim and Lundman, 2004), and CFIR as a coding framework. Authors AH and CV read all the interview transcripts several times to gain a deep understanding of the texts and analysed two interviews together. AH drew up a coding scheme, and the same procedure was applied to the rest of the interviews. We separated the text into meaning units and coded them with a focus on barriers and facilitators, the perceived utility of RHS-13, and the nurses’ role in the PHCC. Coded meaning units were sorted under CFIR constructs and sub-constructs. Codes were reviewed and compared with the original text to ensure verification and consistency. Finally, to synthesize the data, we divided the meaning units based on whether they were from a low-level or high-level implementation center. The two groups were compared and also studied separately to look for patterns of barriers and facilitators within and between the groups in order to investigate whether the different levels of implementation could be explained by the factors influencing implementation outlined in the CFIR. The analysis process was iterative, with frequent dialog about conflicts, and findings were discussed throughout the process with HA to ensure greater credibility.

## Ethics

Ethical approval was obtained from the Swedish ethical review authority (reg: 2019–01408). Informed consent, including information about the scope of the study and the opportunity to withdraw participation at any time, was signed by all participating nurses.

## Results

### Level of RHS-13 use in the health assessment

The number of conducted RHS-13 assessments was compared with data on the total number of HAs performed at each center (Table 1). The three centres that reported use of RHS-13 in over 50% (65–92%) of the HAs were considered as having a high level of implementation, and the five centres below 50% (0–36%) as low level. The variation in RHS-13 use in the low-level group was considerable.

**Table 1:** Percentage of times when RHS-13 was used in HAs per centre and month. Total number of times RHS-13 was used and total number of HAs in parentheses.

	**Month 1**	**Month 2**	**Month 3**	**Month 4**	**Month 5**	**Month 6**	**Total implementation rate %**
	**% (ratio)**	**% (ratio)**	**% (ratio)**	**% (ratio)**	**% (ratio)**	**% (ratio)**	
**Unit 6**	61 (17/28)	100 (19/19)	100 (30/30)	86 (24/28)	100 (22/22)	70 (20/28)	92
**Unit 5**	67 (2/3)	100 (5/5)	100 (4/4)	100 (10/10)	60 (6/10)	70 (7/10)	80
**Unit 1**	55 (6/11)	64 (7/11)	33 (5/15)	56 (5/9)	81 (21/26)	86 (12/14)	65
**Unit 4**	44 (12/27)	36 (4/11)	13 (2/15)	29 (2/7)	29 (5/17)	45 (15/33)	36
**Unit 3**	6 (1/15)	30 (6/20)	67 (8/12)	21 (3/14)	39 (13/33)	20 (5/25)	30
**Unit 2**	44 (7/16)	41 (9/22)	0 (0/9)	25 (1/4)	8 (3/36)	24 (8/34)	23
**Unit 7**	0 (0/56)	0 (0/52)	0 (0/44)	0 (0/43)	0 (0/15)	0 (0/40)	0
**Unit 8**	0 (0/8)	0 (0/0)	0 (0/7)	0 (0/16)	0 (0/8)	0 (0/63)	0

### Documented reasons for exclusion of RHS-13 from the health assessments

Table 2 shows the nurses’ documented reasons for excluding RHS-13 during the implementation period. The most common reasons were language barriers and insufficient time. Reasons for exclusion differed among the two groups.

**Table 2:** Nurses’ documented reasons for excluding RHS-13 from the HA (high = group with high-level use of RHS-13, low = group with low-level use of RHS-13).

Reasons to exclude RHS-13 from the HA		High	Low
Language barrier	RHS-13 not available in patient's spoken language	3	12
	Illiterate patient	27	18
	No interpreter available	1	8
Total		31	38
Insufficient time	Patient arrives late	3	8
	Other concerns the patient needed help with	3	5
	Insufficient time for other reason	7	34
Total		13	47
Patient's need	The patient has spoken a lot about how she/he feels – judged to be too difficult with repetition	3	5
	Family present	0	3
	Patient declined RHS-13	0	8
Total		3	16
Other		3	6
Total number of exclusions		50	107

### Barriers and facilitators

Nurses from all eight centres participated in the interviews. The results are captured under those CFIR categories that were identified as barriers and facilitators for use. When appropriate, the differences between centres with low-level and high-level implementation are described. When appropriate, reported reasons for exclusions (Table 2) are described, combined with interview data (Table 3).

**Table 3:** Overview of identified facilitators for and barriers to RHS-13 use in the groups with high-level and low-level implementation categorized by CFIR domains.

CFIR Domain	CFIR Category	Facilitator or Barrier	High	Low	Explanation of facilitators and barriers
Innovation domain	Innovation design	Barrier	x	x	Lack of translation in specific languages
	Innovation evidence base	Barrier		x	Perceived low face-validity of RHS-13
		Facilitator	x	x	Advantages with a validated questionnaire for this population
	Innovation relative advantages	Facilitator	x	x	Enables the patient to open up about traumatic events, complements the clinical interview
Inner setting	Available resources: time	Barrier	x	x	Insufficient time allocated for integration of RHS-13 into the HA
	Access to knowledge and information	Barrier		x	Nurses with little experience needed more training
Outer setting	Partnership and referrals	Barrier	x	x	No systematization of use of RHS-13 as a base for referral
		Facilitator		x	RHS-13 cut-off points used as a base for referrals
Individuals’ domain and characteristics	Patient's needs, motivation, capability	Barrier	x	x	Some patients not motivated. Not available in some patients’ languages
	Nurses’ needs, motivation, capabilities	Barrier	x		Nurses felt RHS-13 was redundant
		Facilitator	x	x	Nurses made efforts to fit RHS-13 into the HA. Advantages having an objective assessment instrument.

CFIR = Consolidated Framework of Implementation Research (Damschroder et al., 2022)

### Innovation domain

This domain explains how the innovation characteristics influenced the implementation.

### Innovation design

Innovation design was both a barrier and a facilitator for the use of RHS-13. Nurses reported issues related to the available languages and the questionnaire's design. The lack of translation into certain languages was viewed as an important barrier to using RHS-13 in the HAs among both high- and low-level implementers.

*Occasions when I don't use [RHS-13] are when it's not available in their language and the patient might not speak English. So that's also a reason not to use it. (I. 6)*

The symbols of filled jars as response alternatives, developed for illiterate individuals (described in the methods section), were perceived as confusing to the patients and thus a barrier to use. Nurses in both high and low implementation centres excluded RHS-13 for illiterate patients, while a few attempted to use it with an interpreter, which was found to be challenging and suboptimal. RHS-13 compatibility with the HAs was perceived to vary, depending on patients’ literacy levels. Language barriers and illiterate patients was also the most common reason to exclude RHS-13 among all centres using the questionnaire (Table 2).

*Because if the person can't read and write, there's no point in bringing out the RHS. It's possible, it's theoretically possible, to translate, but in terms of time, we don't have room for me to sit with an interpreter and translate all the questions. (I. 2)*

On the other hand, in the high-level centres it was stated that the availability of RHS-13 in many languages contributed to improving access to assessment and care.

Regarding the design, some nurses said that it would have been helpful if RHS-13 was digital, both to send out prior to the HA to save time during the visit and to save administrative time by not having to fill in the cut-offs manually.

### Innovation evidence base

The validity of the RHS-13 instrument was questioned by the low-level centres, and especially by the nurses in the two centres with no reported use. They were sceptical about whether it could facilitate the detection of mental illness, mistrusted its effectiveness, and believed in their own capacity to assess mental health without objective instruments.

*I mostly thought it was a bit difficult because anyone could have pain in muscles or bones and joints for example, um, many of these people have fled from different places, they have a lot of thoughts, they feel helpless, but that doesn't mean they feel bad. (I. 8)*

This was discussed in relation to the patients’ cultural background; for example, that symptoms can have different meaning in different cultures, making interpretation of the results difficult. One nurse described a situation in which her colleagues (with a background elsewhere than Sweden) looked at RHS-13 with their own “cultural-background glasses” and thought that the cut-offs were misleading and ill-matched. This led to low motivation to use it in HAs.

*To cry easily might signify different things and how common it is might vary in different cultures, without meaning you feel very distressed. So, they [the patients] might think the responses are a bit complicated in relation to their actual feelings. (I. 8)*

Some nurses described discrepancies between their own perceptions of a patient's mental status, drawn from the clinical interview, and the RHS-13 cut-off points. For instance, a few expressed concern when some patients got low cut-off points when they described problems they were experiencing, and the other way around.

*But it can also be the case that a patient rates himself very low on RHS, but when you talk to the patient […], and there's so much more behind it. (I. 6)*

Some nurses said that a few items in RHS-13 were difficult to understand in relation to mental-health status.

*The one [item in RHS-13] I notice a lot of people have problems with, it's this one about feelings […] “if you feel loving feelings” or something like that. Some have a little difficulty with it, many ask about it. And I think twice I've had men who've asked [about it] because they've had erection problems. (I. 2)*

On the other hand, it was perceived as positive that a validated questionnaire was finally being introduced for this group, in line with the use of standardized measurements for the detection of mental illness for the general population in primary healthcare.

*I mean, I work at the normal health center where we have a lot of standardized questionnaires, so, it's like now finally we have one we know is validated for this patient group. (I. 1)*

### Innovation relative advantages

RHS-13 was viewed as a complement to the clinical interview in the HA and as contributing to a more comprehensive assessment of mental-health status. It was perceived as specifically helpful for refugees, a group that experiences trauma and suffers from PTSD to a greater extent than other migrant groups.

*It's allowed them to express themselves more in contrast to if I had asked questions [about their mental health]. It's become more profound. (I. 4)*

RHS-13 was perceived as helping to initiate a conversation about mental health and that it was beneficial for patients who had difficulties expressing themselves. It was supportive in motivating patients to accept psychosocial support and as facilitating referrals to psychological specialist care among all the high-level centres and one low, although not among the other centres. Both high and low implementers perceived RHS-13 as helping patients feel comfortable discussing sensitive topics, such as trauma and mental health.

*I think the instrument is really good for continuing to talk to the patient. Sometimes it can be like an icebreaker if they don't talk so much themselves based on the questions I've asked them [in the clinical interview]. For example, like that question […] “do you cry easily, I can see you've filled… what do you think it could be due to?” and then it's usually like running water. (I. 1)*

A few nurses in the low-level centres perceived RHS-13 as too shallow to address mental illness, and some felt that most of the questions were already covered by the clinical interview and therefore RHS-13 was redundant. On the other hand, nurses in the high-level centres felt that the mental health module in the routine HA was too shallow, and that RHS-13 contributed to a profound assessment. Moreover, they explained that patients felt that using a questionnaire addressed issues properly and that it was a validation of their emotions. This was highlighted as a positive factor compared to the clinical interview.

According to some nurses, in both implementation groups, RHS-13 did not offer significant advantages for either the patient interaction or the overall HA in relation to the time it consumed.

### Inner setting

This domain explains the inner setting characteristics that influenced the implementation.

### Available resources: time

Available time was a key factor influencing the use of RHS-13 in both high- and low-level implementation centres; insufficient time to complete the questionnaire and discuss the results was a barrier. For instance, if the patient arrived late, or if other issues required a lot of attention during the HA, there was limited time to assess the patient's mental health status, either with or without RHS-13. Additional time had not been allocated for its incorporation into the HA and a general opinion was that the nurses felt pressured to include all elements, including RHS-13, within the set timeframe for the HA.

*The problem is time, so I can't push this patient either. Sometimes they want to tell me a lot […]. But I don't have time. (I. 7)*

One reason for not introducing RHS-13 to a patient was lack of time in relation to the patient's capabilities or needs (e.g., level of literacy, large family). With large families, some nurses felt stressed about administering RHS-13 to all eligible family members due to the additional time it would take, while others did not see families as a problem.

In one low- and two high-level centres, nurses had the autonomy to manage their time independently and could adjust the HA timeframe according to the requirements. However, the other nurses had little opportunity to allocate their time, and if a health center was pressured with staff shortages the nurse had to prioritize the overall work and sometimes shorten the timeframe for HAs.

*Right now, with the time pressure, we don't have any nurses in general at the health center, so today I do pretty much everything, I have one more colleague but otherwise it's like, a lot like that, I don't just do HAs, and to add another component that you have to catch up with, it can sometimes be a bit stressful. (I. 5)*

Insufficient time, together with language barriers, were documented as the primary reasons for excluding RHS-13 in terms of the exclusion criteria (Table 2).

### Access to knowledge and information

Nurses new to working with HAs asked for repeated training and guidance on when to use RHS-13 during HAs and explanations of its purpose. More experienced nurses in the low-level centres asked for clarity on score implications.

*More information so that I know how to relate to [the scores] and the questions are a bit […] strange, or it could go wrong, then quite a lot still ends up as my individual responsibility to interpret, even though it says like this with numbers, it doesn't really work that way, I think. (I.3)*

They highlighted the need for more training on addressing trauma and mental-health issues in general. Experienced nurses from both high- and low-level implementation centres found the support and guidance provided in the educational meeting where RHS-13 was introduced to be both adequate and sufficient.

### Outer setting

This domain describes the outer setting in which the PHCCs conducting HAs exist and how it might influence implementation.

### Partnership and connections (referral pathways)

The centres referred patients to different psychiatric clinics offering psychosocial support. The RHS-13 cut-off score was sometimes used as a basis for referral to psychiatric care, yet it did not contribute to a systematization of referral pathways to such care. For instance, if a patient was reluctant, the nurses avoided referring them. Nonetheless, the nurses who frequently used RHS-13 perceived that its implementation led to an increase in detecting mental illness during the HA.

*I make more referrals, yes, I think I identify more. Before that [RHS-13], not many people talked about mental illness. (I. 2)*

Nurses noted awareness of the perceived benefits of RHS-13 at two referral psychiatry clinics. Irrespective of RHS-13, referral clinics often redirected patients back to the PHCC. Additionally, at one low-level center, using it led to the implementation of internal guidelines and standardized pathways for referrals. However, although it did not influence when they booked patients to the PHCC's psychotherapist, nurses felt that involvement of the psychotherapist in the educational meeting would assist in the use of RHS-13.

*And I probably would have liked to have the therapist involved in that. (I. 4)*

### Individuals’ domain

*Innovation recipients: patients’ capabilities, motivations, opportunities, and needs*

Several aspects related to patients’ needs and motivations were a barrier to use. For instance, reluctance to address mental health due to stigma and not wanting help despite high scores in RHS-13. Other barriers were low motivation among those who had been in Sweden for a long time and patients’ fear of consequences in psychiatric care. Some nurses in the low-level implementation centres perceived that, in some cultures, mental illness is dealt with and managed within the family, without involvement from healthcare, and therefore felt low motivation to use RHS-13.

*Whereas in other cultures, maybe you take care of it yourself or in the family, that you support each other and how you see the role of care and how bad you have to feel to seek help. (I. 3)*

A common perception across both high- and low-level centres was that it was inconvenient to use RHS-13 with patients who showed little interest in addressing mental health, or with those concentrating on positive aspects and the future. At times, the questionnaire was perceived as a routine task, viewed as something that needed to be checked off from a list.

*It feels more like they're doing schoolwork somehow; I don't know. It doesn't feel emotional… I met a man who didn't want to, he looked through it and said “I don't want to, this is too hard” and that's how it was, but otherwise, I rarely meet anyone who expresses feelings in it the context [of the HA]. Even though we deal with difficult questions. (I. 10)*

Some patients perceived the HA and RHS-13 as obligatory, despite being informed that it was optional.

*Everything is explained that it's completely voluntary […] but I can still sometimes perceive that the patients feel a demand on themselves that they should do it, because they think it's [a requirement] from the Migration Agency's side or based on the fact that there's still a demand. (I. 5)*

*Innovation deliverers: nurses’ capabilities, motivations, opportunities, and needs*

Capability and motivation among nurses were both a facilitator and barrier to using RHS-13. Both in high- and low-level centres, RHS-13 contributed with positive aspects and nurses appreciated having an objective tool facilitating the mental-health assessment*:*

*I like instruments like this, it makes it easier, uh, yes, I'm positive about it. (I. 4)*

Barriers described under other domains above were: time, families, scepticism about its effectiveness, and redefining its purpose, all of which influenced the motivation to use RHS-13, specifically in the low-level centres. Their opportunity was also influenced by other on-going studies, a larger influx of migrants than anticipated, and the on-going Covid-19 pandemic.

*[I need] more justification about why [RHS-13] is good, or how to think about using it, what kind of effect it should have. So that you understand more, because everyone understands, here's a form to be filled in, but what's the purpose? (I. 3)*

In one low-level and all the high-level centres, this was less problematic because they perceived RHS-13 as facilitating the HA by complementing it.

Despite the extra time requirements and occasional stress related to its use, the majority intended to use RHS-13 and saw its potential to improve mental-health assessments. Efforts were made to optimize use and save time; for example, one nurse sent RHS-13 with the HA invitation so that patients could fill in the questionnaire before their visit, instead of integrating it during the clinical interview.

*No, always. It has [X] said, it must always be done. Provided there's time and the patient want to, of course, but… But it's always part of what you go through. (I. 10)*

## Discussion

This study has examined the implementation of RHS-13 in the HAs for forced migrants in Stockholm Region and has identified barriers and facilitators for its use. This contributes to knowledge about using standardized measurements for assessing mental illness in a forced migrant population. Most studies have tested RHS-13 in immediate reception centres, where most individuals are accommodated for months with limited freedom of movement (Fontana et al., 2023). Our study broadens the understanding of how RHS-13 can be used for addressing mental illness in routine care in a resettlement context and provides insights into contextual aspects that need to be considered for implementation.

We found that the use of RHS-13 varied between centres, resulting in two groups: high-level and low-level implementation centres. Factors related to the inner context and to the design of the questionnaire influenced its use. There were more barriers to implementing RHS-13 represented in the low-level group and its use was affected by language barriers, limited time, and lack of trust in its clinical benefit.

### Language barriers

There were two types of language barriers: (i) RHS-13 was not available in all patient languages (meaning that an interpreter was needed to translate the questions), and (ii) illiterate patients could not answer the questionnaire despite it being in their language. Although all the centres use interpreters for HAs, the use of RHS-13 with an interpreter was not compatible. In theory, the nurse could guide a patient through the questionnaire with the help of interpreters; however, during Covid-19, interpreters were usually working via telephone. This, in combination with limited time to introduce the interpreter to the questionnaire, made it difficult to use RHS-13 when interpretation was required. Previous studies have shown positive results when addressing mental health using self-assessment questionnaires with interpreters who are trained and have been introduced to the questionnaire (Magwood et al., 2022; Kiselev et al., 2020; Wylie et al., 2018; Winkler, 2015). However, using interpreters may introduce bias and errors, especially with non-translatable or culturally nuanced concepts. Therefore, the effective use of interpreters requires preparation, relationship-building, and debriefing among healthcare providers to address misunderstandings and linguistic complexities, something that was not done in the context of this study. This may explain why nurses perceived it as less than optimal to use RHS-13 with an interpreter (Willey et al., 2020; Winkler, 2015; Heath et al., 2023; Bauer and Alegría, 2010).

### Limited time

Lack of time, and generally scarce resources, together with a heavy and complex workload, are well-known barriers to implementing innovations in primary healthcare settings (Mather et al., 2022; Van Ginderdeuren et al., 2019). However, the results of this study add to the picture of a heavy workload by showing how degrees of autonomy regarding how work is organised can also influence the perception of time. Limited time was more common in the low-level implementation group, where the nurses also perceived fewer opportunities to organize and regulate their work time, compared to nurses in the high-level centres. This indicates that this barrier can be reduced by altering the ways in which work is organized, as demonstrated in other studies, and that available resources influence how nurses set priorities, with higher levels of autonomy increasing work satisfaction (Hendry and Walker, 2004; Holmér et al., 2023).

### Perceived fit and acceptance of RHS-13

While psychometric reliability and validity in RHS-13 has been demonstrated (Magwood et al., 2023; Hollifield et al., 2016; Bjärtå et al., 2018), less is known about its acceptance and perceived fit among healthcare staff in the clinical routine. Despite the fact that it was introduced in the same way to all centres, some nurses questioned its validity and valued their own clinical judgement more highly. Nurses were also influenced by patients’ motivations and needs, such as stigma associated with mental-health issues or just not wanting to use the questionnaire. Clinicians’ perceptions of an intervention are not necessarily connected to actual evidence but are also associated with how acceptable and feasible the intervention is and its perceived benefits for their patients, which in turn can be affected by available resources such as time. The fact that mistrust was greater in the low-level implementation centres could be influenced by the limited time and the nurses’ lack of autonomy to organize their own work, perhaps leading to less time to familiarize themselves with RHS-13 and its possible benefits.

### Perceived advantages

Our findings suggest that RHS-13 was perceived as a valuable supplement to the clinical interview, but not for everyone receiving an HA. This is in line with other studies, suggesting that a combination of methods is preferred, such as self-assessment questionnaires combined with clinical interviews (Sheth et al., 2023; Wylie et al., 2018). RHS-13 did not lead to the systematization of referrals for those needing more care. Screening only works if it improves access to care for those screening positive. The nurses described barriers in referring to secondary care, with referrals often being sent back to the PHCC. It has been established that migrants in high-income countries utilize less psychiatric care than the majority population except for compulsory care (Hollander et al., 2020; Morgan et al., 2005). In the interviews, the nurses requested more involvement from psychotherapists working at healthcare centres in the implementation of RHS-13, and more training on understanding its cut-off scores and how to use them for referrals. For nurses, it can be ethically challenging to carry out screening without knowing if it will lead to the relevant healthcare (CDC 2015; Sheth et al., 2023). If integrated properly with referrals, RHS-13 cut-off scores could guide decision-making for further care and facilitate a seamless care transition to avoid disruption in the care pathway. Integrating RHS-13 results in a standardized way can enhance assessments, not just by making them more consistent and reliable, but also by ensuring that they are quantifiable and comparable. If used effectively, standardized methods can reduce the time needed to assess migrants and promote the equal and early detection of mental illness (Magwood et al., 2022; Rhema et al., 2020). However, this requires clear pathways for referral.

### Implications for implementation and recommendations

All the PHCCs in this study received the same implementing strategies, but the use of RHS-13 varied, suggesting a need to tailor these strategies to local needs. The Behaviour Change Wheel's three cornerstones (Michie et al., 2011) help in assessing the need for targeted implementation strategies: Enhancing capability involves training and education, as indicated by the nurses’ requests for additional guidance on interpreting RHS-13 scores; Opportunity means having a supportive structural environment, such as adequate time and resources, including interpreters for illiterate patients and autonomy to organize and regulate worktime; Motivation, influenced by a clear understanding of clinical relevance, varied among nurses, and while RHS-13 was perceived as valuable for mental-health assessment, doubts about its effectiveness were expressed.

For the future, assessing barriers and facilitators prior to implementation of RHS-13 and selecting appropriate implementation strategies based on this assessment is recommended. The Behaviour Change Wheel or the CFIR-ERIC matching tool (Waltz et al., 2019) can be valuable for matching strategies to existing barriers. Based on the findings in this study, key steps for implementation could include management to enable the planning of nurses’ autonomy concerning work organization and time, involving administrators in planning and educational meetings, improving interpreter services, especially for illiterate patients, and strengthening referral processes prior to implementation. Additionally, it is critical to include local psychosocial staff and clearly communicate the purpose and benefits of RHS-13, particularly cut-off score interpretation and follow-up, in educational meetings and materials, and within collaborative learning.

### Methodological discussion

The mixed-methods approach, combining RHS-13 usage, exclusion reasons, and nurses’ experiences of barriers and facilitators, provided comprehensive insights into RHS-13 implementation in routine practice. In terms of dependability, the mixed expertise in both migration-related health consequences and implementation among the authors enhanced and broadened the discussion of the analysis.

To our knowledge, this study is among the first to use the new updated version of CFIR 2.0 to investigate barriers to and facilitators for implementing an innovation in routine care. It adds important insights into the constant theory-constructing of CFIR 2.0 by applying the framework to implementing healthcare methods in migration and health research. This in line with the CFIR teams ambition to develop intersectoral approaches and apply the framework to research concerning populations from low- and middle-income countries (Damschroder et al., 2022).

The study findings reveal overlapping CFIR categories. For example, insufficient time can be attributed to both inadequate resource allocation, including the lack of designated time for implementation, and the overarching work structure for the HA, irrespective of whether RHS-13 is integrated into the HA. Despite this type of methodological challenge for categorizing and examining the interview data, these insights accurately represent the multifaceted and complex organizational reality of PHCCs, which often influences the adoption of innovations and restricts their full implementation.

All eight PHCCs performing HAs in Stockholm Region participated in the study. Despite this, the study population was small due to the low number of staff engaged in this part of the healthcare sector. This could challenge the trustworthiness of the qualitative data, in terms of the representativeness of the participants. On the other hand, representation from all the PHCCs guarantees different perspectives, as they meet different patients and have different prerequisites regarding how the inner setting is organized, as well as different amounts of control over their time and workload. Moreover, rich data material was collected and analysed. Categories were replicated, which demonstrates completeness and verification (Elo et al., 2014).

Our study was conducted during the Covid-19 pandemic. HAs continued on-site as usual, but with more interpreters on the telephone instead of in person. Most of the participants did not raise Covid-19 as a disturbing factor; however, additional information needed to be disseminated, which encroached on their time. Furthermore, educational meetings had to be held online due to Covid-19 restrictions, in contrast to the new nurses’ request to receive repeated training on RHS-13 on site. On-site training might have resulted in higher levels of implementation of RHS-13 in the HA.

Another limitation is the relatively short evaluation period. Achieving a high level of implementation requires repeated practice in order to normalize use and build capacity, which in turn can lead to greater motivation and then greater use. In line with this, RHS-13 is now included in the educational meetings for new and existing centres performing HAs and is recommended as a supplementary tool in the HA in Stockholm.

## Conclusion

RHS-13 was considered to have advantages over conventional methods for addressing mental health in the HA, but not for screening the whole population. It also contributed towards standardizing the mental-health assessment, something that did not exist before this study. The implementation of RHS-13 was influenced by multiple factors at different levels. Identifying context-based implementation strategies and addressing issues of language and time resourcing as well as nurses trust in the tool's utility are recommended to enhance the use of RHS-13.

## Funding

The study was part of a quality assurance project funded by Stockholm Region (grant 2018–0034, project 1513). The funding did not influence the study design, data collection, analysis, interpretation of data, or preparation of the manuscript. Open Access funding was provided by Karolinska Institutet.

## CRediT authorship contribution statement

**Ana Hagström:** Writing – review & editing, Writing – original draft, Project administration, Methodology, Investigation, Formal analysis, Conceptualization. **Henna Hasson:** Writing – review & editing, Funding acquisition, Formal analysis, Conceptualization. **Anna-Clara Hollander:** Writing – review & editing, Formal analysis. **Carl Vahtra:** Investigation, Formal analysis. **Sara Delilovic:** Writing – review & editing, Methodology. **Hanna Augustsson:** Writing – review & editing, Writing – original draft, Methodology, Formal analysis, Conceptualization.

## Declaration of competing interest

The authors declare that they have no known competing financial interests or personal relationships that could influence the work reported in this paper.

## Appendix Supplementary materials

Image, application 1

Image, application 2

Image, application 3

Image, application 4

Image, application 5

Image, application 6
