# Supplementary material for: “Sometimes it can be like an icebreaker”: A mixed method evaluation of the implementation of the Refugee Health Screener-13 (RHS-13)
Source: J Migr Health. 2024 Jul 15;10:100243. doi: 10.1016/j.jmh.2024.100243 (PMC11365362; doi:10.1016/j.jmh.2024.100243)
Supplement: Supplementary file 1 [file mmc1.docx]

*Supplementary material 1*

Table 1. Description of the eight participating centres: number of nurses conducting health examinations and their responsibilities at each centre

|  | Nr of nurses | Responsibilities |
| --- | --- | --- |
| Centre 1 | 1 | Mainly health examinations |
| Centre 2 | 1 | Mainly health examinations |
| Centre 3 | 2-3* | Health examinations and other patient responsibilities in primary health centre |
| Centre 4 | 3 | Health examinations and other patient responsibilities in primary health centre |
| Centre 5 | 1 | Mainly health examinations |
| Centre 6 | 1 | Mainly health examinations |
| Centre 7 | 2 | Mainly health examinations |
| Centre 8 | 2-3* | Health examinations and other patient responsibilities in primary health centre |

*Two nurses mainly conducted the health examinations but at some time points one extra nurse conducted health examinations
