# Supplementary material for: “Sometimes it can be like an icebreaker”: A mixed method evaluation of the implementation of the Refugee Health Screener-13 (RHS-13)
Source: J Migr Health. 2024 Jul 15;10:100243. doi: 10.1016/j.jmh.2024.100243 (PMC11365362; doi:10.1016/j.jmh.2024.100243)
Supplement: Supplementary file 2 [file mmc2.docx]

## Interview guide. Implementation of RHS-13 in the health assessment for asylum seekers and new arrivals in Region Stockholm (translated from Swedish)

Health assessment = HA

**Prequestion:** Did you and your collagues use RHS-13 during th past six months? ***To the interviewer:*** *If the answer is yes, proceed to question 1. If the answer is no, proceed to*  ***question A*** *immediately after question 2. (Rissne health centre has not used – go directly to A with them).*

**Introduction**

1. Tell us about your professional role and your work here at the asylum reception
2. Tell us about how a HA conversation works from conversations about the psychosocial life situation, the assessment with RHS and the referral to a doctor and counsellor? Even when RHS has not been used

**Utility and usefulness**

1. Can you tell us about your experiences with a mental health screening instrument?
2. What has worked well?
3. What motivates you to use RHS-13 in the health conversation?
4. If any, what benefit do you feel that RHS-13 has had for the patient?

**Worksituation and collaboration**

1. How has the introduction of the new way of working with RHS-13 affected your work situation? What has it meant to you in the meeting with the patient?
2. In what way are you influenced by your colleagues to use RHS-13 in the HA? *Probe: peer pressure, social support, work environment and conflicts*
3. How does the use of RHS-13 affect your allocated time for the HA?
4. What are your experiences regarding collaboration with doctors and other professions at the asylum reception regarding RHS? How has this affected referral, *please elaborate.*
5. What is offered to the patient if mental illness is identified with RHS-13? *Please elaborate*
6. How do you feel about using RHS-13 without always being able to offer follow-up? *Probe: Some people find it difficult to screen for mental well-being without having an action plan, be able to offer psychological support, referral to a psychiatrist is available in some cases)*
7. What do you think is important for mental health screening to work? What prerequisites are required?

**Characteristics of the intervention and referrals**

1. How does RHS-13 compare to other approaches to identifying signs of mental illness in the HA?
2. In what way do you feel that RHS-13 is consistent with your "general impression of the patient's mental illness? Does it capture the right things?
3. What are your experiences of referring patients with mental illness? Is there any difference now that you are using RHS-13, if any, for whom do you think RHS-13 makes a difference? *Probe, nurse or patient?*

**Motivation**

1. Do you think that you/your colleagues have sufficient knowledge about why RHS-13 is used in the HA? *Probe: Knowledge of why it is used, information about why it is used by different actors; the client, management, others*
2. Does RHS-13 add anything to HE? Develop
3. Is there a need to introduce RHS-13 in the health assessment?
4. Do you/your colleagues have the intention to always use RHS-13? In certain situations?

**Adaptation and the Inner Context**

1. How well does RHS-13 fit into existing UH? *What has it been like to work with an interpreter when RHS-13 is used to screen mental well-being, develop.*
2. How do you experience the screening instrument? Feasability with the HA? *Probe: Is it an appropriate context?*
3. Do you feel that working with RHS-13 is a priority? Have other commitments been given higher priority? *Support for the interviewer: In the work situation, we always have to prioritize, in a stressful situation, some things can be prioritized higher than others*

**Adaptations and compliance**

1. During the test period, you have documented level of use (statistics on use). Specific question on level of implementation to each nurse regerading her documentation. Can you tell us a little bit more about that?
2. Tell us about the situations in which you have used and not used RHS-13 in HA? *Develop*
3. What challenges have you experienced with using a screening instrument in the conversation with the patient? (ex: linguistic, cultural, etc.)
4. Have you used RHS-13 according to the instructions you received at the start or have you made any adjustments to how RHS-13 should be used in HA? *Probe:specific situations,e.g. all patients should be screened. Barriers? Certain groups different barriers? Factors influencing the adoption of RHS-13?*
5. Is it hard to remember to use RHS-13 in conversation? If so, why do you think and what would help you and your colleagues to remember to use RHS-13?

**Process and support**

1. How do you perceive the attitude and role of management in relation to the introduction of RHS-13? *Is the management aware that RHS-13 is being tested and used in the HA?*
2. What kind of support have you received for using RHS-13? *Probe: From your management team and from other actors? For example, the Health Care Administration or others?*
3. What are your prospects for introducing screening for mental illness permanently?
4. Do you see any concerns/risks with screening?
5. How could the implementation of RHS have been done differently from your perspective?

***Finally, is there anything I've forgotten to ask you that you'd like to bring up?***

**Additional questions for centres with inconsistent use**

1. Usually when innovations or new methods have not been fully used it is due to some particular reason, what is this thea reason in this case with RHS-13 according to you?
2. In your opinion, is there a need to introduce RHS-13 in HA?
3. Could anything have been done differently when introducing RHS-13 in HA that could have made it easier for you to use RHS-13?
4. Have you requested support for implementation during the period RHS-13 was used used?
5. What changes need to be made in order for you to be able to start using RHS-13 in HA?
6. ***Finally, is there anything I've forgotten to ask you that you'd like to bring up?***
