# Supplementary material for: “Sometimes it can be like an icebreaker”: A mixed method evaluation of the implementation of the Refugee Health Screener-13 (RHS-13)
Source: J Migr Health. 2024 Jul 15;10:100243. doi: 10.1016/j.jmh.2024.100243 (PMC11365362; doi:10.1016/j.jmh.2024.100243)
Supplement: Supplementary file 4 [file mmc4.pdf]

PATHWAYS TO WELLNESS

Integrating Refugee Health and Well-Being

# Refugee Health Screener-15 (RHS-15)

## English Version

*Bilingual versions of the RHS-15 have been translated by an iterative process involving experts in the field, professional translators, and members of the refugee community so that each question is asked correctly according to language and culture. The English text is provided for reference only; using the English alone negates the sensitivity of this instrument.*

**DEMOGRAPHIC INFORMATION**

Name: ______________________ Date of Birth: ______________________

Gender: ____________ Date of Arrival: ____________ Health ID: ____________

Administered by: ______________________ Date of Screen: ______________________

Developed by the *Pathways to Wellness* project and generously funded by Robert Wood Johnson Foundation, Bill and Melinda Gates Foundation, United Way of King County, Medina Foundation, The Seattle Foundation, Boeing Employees Community Fund and M.J. Murdock Charitable Trust.

© 2013 Pathways to Wellness: Integrating Refugee Health and Well-Being

Pathways to Wellness: Integrating Refugee Health and Well-Being is a project of Lutheran Community Services Northwest, Asian Counseling and Referral Service, Public Health Seattle & King County, and Michael Hollifield, M.D. of Pacific Institute for Research & Evaluation. For more information, please contact The *Pathways* Project at 206-816-3253 or pathways@lcsnw.org.

ID#

DATE

# REFUGEE HEALTH SCREENER-15 (RHS-15)

PATHWAYS TO WELLNESS
Integrating Refugee Health and Well-Being

**INSTRUCTIONS:** Using the scale beside each symptom, please indicate the degree to which the symptom has been bothersome to you over the past month. Place a mark in the appropriate column. If the symptom has not been bothersome to you during the past month, circle "NOT AT ALL."

| SYMPTOMS | NOT AT ALL | A LITTLE BIT | MODERATELY | QUITE A BIT | EXTREMELY |
|---|---|---|---|---|---|
| **1. Muscle, bone, joint pains** | 0 | 1 | 2 | 3 | 4 |
| **2. Feeling down, sad, or blue most of the time** | 0 | 1 | 2 | 3 | 4 |
| **3. Too much thinking or too many thoughts** | 0 | 1 | 2 | 3 | 4 |
| **4. Feeling helpless** | 0 | 1 | 2 | 3 | 4 |
| **5. Suddenly scared for no reason** | 0 | 1 | 2 | 3 | 4 |
| **6. Faintness, dizziness, or weakness** | 0 | 1 | 2 | 3 | 4 |
| **7. Nervousness or shakiness inside** | 0 | 1 | 2 | 3 | 4 |
| **8. Feeling restless, can't sit still** | 0 | 1 | 2 | 3 | 4 |
| **9. Crying easily** | 0 | 1 | 2 | 3 | 4 |

Developed by the Pathways to Wellness project and generously funded by Robert Wood Johnson Foundation, Bill and Melinda Gates Foundation, United Way of King County, Medina Foundation, The Seattle Foundation, Boeing Employees Community Fund and M.J. Murdock Charitable Trust.

© 2013 Pathways to Wellness: Integrating Refugee Health and Well-Being

Pathways to Wellness: Integrating Refugee Health and Well-Being is a project of Lutheran Community Services Northwest, Asian Counseling and Referral Service, Public Health Seattle & King County, and Michael Hollifield, M.D. of Pacific Institute for Research & Evaluation. For more information, please contact The Pathways Project at 206-816-3253 or pathways@lcsnw.org.

ID#

DATE

# REFUGEE HEALTH SCREENER-15 (RHS-15)

PATHWAYS TO WELLNESS
Integrating Refugee Health and Well-Being

The following symptoms may be related to traumatic experiences during war and migration. How much in the past month have you:

| SYMPTOMS | NOT AT ALL | A LITTLE BIT | MODERATELY | QUITE A BIT | EXTREMELY |
|---|---|---|---|---|---|
| **10. Had the experience of reliving the trauma; acting or feeling as if it were happening again?** | 0 | 1 | 2 | 3 | 4 |
| **11. Been having PHYSICAL reactions (for example, break out in a sweat, heart beats fast) when reminded of the trauma?** | 0 | 1 | 2 | 3 | 4 |
| **12. Felt emotionally numb (for example, feel sad but can't cry, unable to have loving feelings)?** | 0 | 1 | 2 | 3 | 4 |
| **13. Been jumpier, more easily startled (for example, when someone walks up behind you)?** | 0 | 1 | 2 | 3 | 4 |

Developed by the Pathways to Wellness project and generously funded by Robert Wood Johnson Foundation, Bill and Melinda Gates Foundation, United Way of King County, Medina Foundation, The Seattle Foundation, Boeing Employees Community Fund and M.J. Murdock Charitable Trust.

© 2013 Pathways to Wellness: Integrating Refugee Health and Well-Being

Pathways to Wellness: Integrating Refugee Health and Well-Being is a project of Lutheran Community Services Northwest, Asian Counseling and Referral Service, Public Health Seattle & King County, and Michael Hollifield, M.D. of Pacific Institute for Research & Evaluation. For more information, please contact The Pathways Project at 206-816-3253 or pathways@lcsnw.org.

ID#

DATE

## REFUGEE HEALTH SCREENER-15 (RHS-15)

**14. Circle the one best response below. Do you feel that you are:**

| | |
|---|---|
| **Able to handle (cope with) anything** | **0** |
| **Able to handle (cope with) most things** | **1** |
| **Able to handle (cope with) some things, but not able to cope with other things** | **2** |
| **Unable to cope with most things** | **3** |
| **Unable to cope with anything** | **4** |

**Add Total Score of items 1–14** ______

**15. Distress Thermometer**

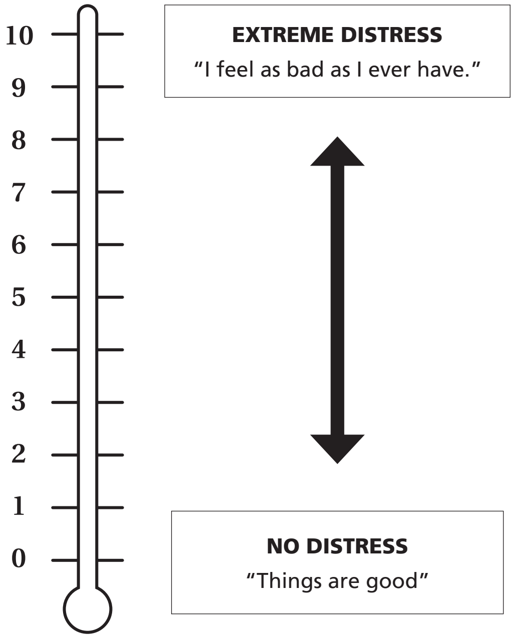


Please circle the number (0–10) that best describes how much distress you have been experiencing in the past week, including today.

**SCORING** SCREENING IS POSITIVE IF: ❶ ITEMS 1–14 IS ≥12 OR ❷ DISTRESS THERMOMETER IS ≥5

**CHECK ONE:** ☐ **POSITIVE** ☐ **NEGATIVE** ☐ SELF-ADMINISTERED ☐ NOT SELF-ADMINISTERED

LEGAL NOTICE 2013 © Pathways to Wellness: Integrating Refugee Health and Wellbeing. Pathways to Wellness is a partnership of Lutheran Community Services Northwest, Asian Counseling and Referral Service, Public Health Seattle and King County and Michael Hollifield, MD of Pacific Institute for Research and Evaluation. All Rights Reserved.
