# Supplementary material for: “Sometimes it can be like an icebreaker”: A mixed method evaluation of the implementation of the Refugee Health Screener-13 (RHS-13)
Source: J Migr Health. 2024 Jul 15;10:100243. doi: 10.1016/j.jmh.2024.100243 (PMC11365362; doi:10.1016/j.jmh.2024.100243)
Supplement: Supplementary file 5 [file mmc5.pdf]

ID# ______________

DATE ______________

REFUGEE HEALTH SCREENER-15 (RHS-15)
IN SWEDISH

PATHWAYS TO WELLNESS

Instruktioner: Ange i skalan vid varje symptom i vilken grad symptomet har besvärat dig under den senaste månaden. Ringa in det alternativ som bäst passar in på dig. Om symptomet inte har besvärat dig under den senaste månaden ska du ringa in "0" som betyder "INTE ALLS"

INSTRUCTIONS: Using the scale beside each symptom, please indicate the degree to which the symptom has been bothersome to you over the past month. Place a mark in the appropriate column. If the symptom has not been bothersome to you during the past month, circle "NOT AT ALL."

**SYMPTOM**<br>**SYMPTOMS**	**INTE ALLS** *(not at all)*	**I LITEN GRAD** *(a little bit)*	**MÅTTLIGT** *(moderately)*	**I HÖG GRAD** *(quite a bit)*	**I MYCKET HÖG GRAD** *(extremely)*
1. **Värk i muskler, skelett eller leder**<br>Muscle, bone, joint pains	0	1	2	3	4
2. **Känt dig ledsen eller nedstämd större delen av tiden**<br>Feeling down, sad, or blue most of the time	0	1	2	3	4
3. **Tänkt för mycket eller haft för många tankar**<br>Too much thinking or too many thoughts	0	1	2	3	4
4. **Känt dig hjälplös**<br>Feeling helpless	0	1	2	3	4
5. **Plötsligt känt dig rädd utan anledning**<br>Suddenly scared for no reason	0	1	2	3	4
6. **Svimfärdig, yr eller svag**<br>Faintness, dizziness, or weakness	0	1	2	3	4
7. **Nervositet eller skakighet inombords**<br>Nervousness or shakiness inside	0	1	2	3	4
8. **Känt dig rastlös, kan inte sitta stilla**<br>Feeling restless, can't sit still	0	1	2	3	4
9. **Lätt att gråta**<br>Crying easily	0	1	2	3	4

Developed by the Pathways to Wellness project and generously funded by Robert Wood Johnson Foundation, Bill and Melinda Gates Foundation, United Way of King County, Medina Foundation, The Seattle Foundation, Boeing Employees Community Fund and M.J. Murdock Charitable Trust.

Production of the Cuban Spanish RHS-15 was made possible by the Kentucky Office for Refugees.

© 2013 Pathways to Wellness: Integrating Refugee Health and Well-Being

Pathways to Wellness: Integrating Refugee Health and Well-Being is a project of Lutheran Community Services Northwest, Asian Counseling and Referral Service, Public Health Seattle & King County, and Michael Hollifield, M.D. of Pacific Institute for Research & Evaluation. For more information, please contact The Pathways Project at 206-816-3253 or pathways@lcsnw.org

ID#

REFUGEE HEALTH SCREENER-15 (RHS-15)
IN SWEDISH

PATHWAYS TO WELLNESS

DATE

Följande symptom kan vara relaterade till traumatiska upplevelser under krig och migration. I vilken grad har du under den senaste månaden:

The following symptoms may be related to traumatic experiences during war and migration. How much in the past month have you:

SYMPTOM<br>SYMPTOMS	INTE ALLS *(not at all)*	I LITEN GRAD *(a little bit)*	MÅTTLIGT *(moderately)*	I HÖG GRAD *(quite a bit)*	I MYCKET HÖG GRAD *(extremely)*
**10. Återupplevt traumat; att du agerat eller känt som om att det händer igen?**<br>Had the experience of reliving the trauma; acting or feeling as if it were happening again?	0	1	2	3	4
**11. Haft kroppsliga reaktioner (t ex svettningar, hjärtklappning) när du blivit påmind om traumat?**<br>Been having physical reactions (for example, break out in a sweat, heart beats fast) when reminded of the trauma?	0	1	2	3	4
**12. Känt dig känslomässigt avtrubbad (t ex varit ledsen utan att kunna gråta, inte kunnat känna kärlek)?**<br>Felt emotionally numb (for example, feel sad but can't cry, unable to have loving feelings)?	0	1	2	3	4
**13. Känt dig lättskrämd, haft lättare att rycka till (t ex av plötsliga ljud eller rörelser)?**<br>Been jumpier, more easily startled (for example, when someone walks up behind you)?	0	1	2	3	4

Developed by the Pathways to Wellness project and generously funded by Robert Wood Johnson Foundation, Bill and Melinda Gates Foundation, United Way of King County, Medina Foundation, The Seattle Foundation, Boeing Employees Community Fund and M.J. Murdock Charitable Trust.

Production of the Cuban Spanish RHS-15 was made possible by the Kentucky Office for Refugees.

© 2013 Pathways to Wellness: Integrating Refugee Health and Well-Being

Pathways to Wellness: Integrating Refugee Health and Well-Being is a project of Lutheran Community Services Northwest, Asian Counseling and Referral Service, Public Health Seattle & King County, and Michael Hollifield, M.D. of Pacific Institute for Research & Evaluation. For more information, please contact The Pathways Project at 206-816-3253 or pathways@lcsnw.org
