# Supplementary material for: “Sometimes it can be like an icebreaker”: A mixed method evaluation of the implementation of the Refugee Health Screener-13 (RHS-13)
Source: J Migr Health. 2024 Jul 15;10:100243. doi: 10.1016/j.jmh.2024.100243 (PMC11365362; doi:10.1016/j.jmh.2024.100243)
Supplement: Supplementary file 6 [file mmc6.docx]

**Self-reported use of the Refugee Health Screener and reasons to exclude RHS-13**

| **Date of visit** | **Gender (M/F)** | **Screened** | **Not screened** | **If not screened – reason why** | | | | | | | | | | |
| --- | --- | --- | --- | --- | --- | --- | --- | --- | --- | --- | --- | --- | --- | --- |
|  |  |  |  | Language difficulties in general | RHS is not available in the correct language | The patient cannot read and interpretation is done over the phone | The patient can read/write but the RHS is not translated into the correct language | The patient has told us a lot about how they feel – it is judged to be too difficult with repetition | Family present | Lack of time - patient arrives too late | Lack of time - other things that the patient needed help with that took up the whole conversation | Lack of time – other | Patient  declines | Other |
|  |  |  |  |  |  |  |  |  |  |  |  |  |  |  |
|  |  |  |  |  |  |  |  |  |  |  |  |  |  |  |
|  |  |  |  |  |  |  |  |  |  |  |  |  |  |  |
|  |  |  |  |  |  |  |  |  |  |  |  |  |  |  |

**Fill in for each individual who attends a health assessment**
